# Serum Albumin–Creatinine Ratio and Anthracycline Cardiotoxicity in Patients with Cancer

**DOI:** 10.3390/jcm14051741

**Published:** 2025-03-05

**Authors:** Onur Baş, Naciye Güdük, Mert Tokatlı, Deniz Can Güven, Necla Özer, Yusuf Ziya Şener, Serkan Akın, Sercan Aksoy, İbrahim Barışta, Fatma Alev Türker, Ömer Dizdar

**Affiliations:** 1 Division of Medical Oncology, Department of Internal Medicine, Faculty of Medicine, Hacettepe University, Sihhiye, 06100 Ankara, Turkeysaksoy07@yahoo.com (S.A.); aturker@hacettepe.edu.tr (F.A.T.);; 2 Department of Internal Medicine, Faculty of Medicine, Hacettepe University, Sihhiye, 06100 Ankara, Turkey; naciyeguduk@hotmail.com (N.G.);; 3 Elazig Fethi Sekin Sehir Hastanesi, Health Sciences University, 23300 Elazig, Turkey; denizcguven@hotmail.com; 4 Department of Cardiology, Faculty of Medicine, Hacettepe University, Sihhiye, 06100 Ankara, Turkey; neclaozer@hacettepe.edu.tr (N.Ö.);

**Keywords:** anthracycline, serum albumin–creatinine ratio, cardiotoxicity, cancer

## Abstract

**Background:** Several studies have suggested that the serum albumin–creatinine ratio (sACR) is a useful marker for the early risk stratification of patients with cardiomyocyte injury. This study aims to evaluate the relationship between sACR and anthracycline-related cardiotoxicity. **Methods:** This study included patients who had received anthracycline-based chemotherapy between 2014 and 2023 and had undergone baseline and follow-up echocardiography after the treatment. The level of sACR was calculated using serum albumin and creatinine values obtained before the chemotherapy. The definition of cardiotoxicity was based on the criteria of the European Society of Cardiology (ESC) for ejection fraction and the American Society of Echocardiography (ASE) for diastolic dysfunction. The patients were categorized into either the high or low sACR group based on the cut-off value obtained from the receiver operating characteristic (ROC) curve analysis. **Results:** In total, 525 patients (159 males, 366 females) were included. Multivariate analysis after adjustment for age, body mass index (BMI), cardiovascular disease, hemoglobin, anthracycline dose, and gender showed that sACR (HR = 1.85% 95 CI 1.12 to 3.06 *p* = 0.016), cardiovascular disease (HR = 1.97% 95 CI 1.08 to 3.61 *p* = 0.027), BMI (HR = 1.86% 95 CI 1.12 to 3.10 *p* = 0.017), and age (HR = 1.02% 95 CI 1.001 to 1.04 *p* = 0.036) were significantly associated with an increased risk of cardiotoxicity. **Conclusions:** This study is the first to show a significant relationship between sACR and cardiotoxicity related to anthracycline use. Routine laboratory tests that are conducted before anthracycline therapy can aid clinicians in identifying high-risk patients who may require closer follow-up or cardioprotective measures.

## 1. Introduction

Albumin is a type of protein that is found in human plasma and is considered to be a significantly stable one. It plays a crucial role in the human body by performing multiple important functions such as maintaining osmotic pressure, binding compounds, and exhibiting antioxidant activity. However, the level of albumin in the serum can be affected by various factors, such as inflammation, malnutrition, and cancer cachexia, leading to a condition called hypoalbuminemia [1]. This condition is a cause for concern, as the concentration of serum albumin is an essential marker used to assess the nutritional status of a person and predict the severity of diseases and chemotherapy-related toxicities. In previous studies, it has been shown that the serum albumin level is a prognostic factor in cancer. Low levels of albumin have been associated with the poor survival of cancer patients [2]. Albumin levels are also important in patients with excessive inflammation, such as burns [3] and acute pancreatitis [4], and hypoalbuminemia is also associated with the risk of infection [5]. However, it is important to note that many factors can interfere with albumin levels, which limits its usage in daily practice on its own.

Serum creatinine is a crucial marker that plays a significant role in assessing the overall health status of cancer patients. It is a waste product that is produced by the muscles during metabolism, and its levels are directly linked to the nutritional status and the overall muscle mass of the patient. Low levels of creatinine are frequently observed among cancer patients, which is a major concern, as it can lead to increased toxicity from chemotherapy and poorer survival rates. Moreover, impaired renal function at the time of cancer diagnosis has been associated with higher morbidity and mortality rates. Therefore, the level of serum creatinine is an essential marker used to assess the patient’s performance status and the course of the disease. Several studies have highlighted the importance of serum creatinine levels in predicting the outcomes of different types of cancer. For instance, a study conducted by Panotopoulos and his colleagues found that serum creatinine levels are a significant prognostic factor in patients with liposarcoma [6]. Similarly, serum creatinine levels are a reliable predictor of overall survival in patients with colon cancer [7]. In cases of an elevated systemic inflammatory response, such as acute pancreatitis and sepsis, creatinine levels are also of significance [8,9]. In summary, monitoring serum creatinine levels is a crucial aspect of cancer care, as it helps assess the patient’s overall health status, predict the course of the disease, and determine the appropriate treatment plan.

The serum albumin–creatinine ratio (sACR) has been demonstrated to be a significant predictor of hospital mortality in patients with pancreatitis [10]. Furthermore, Turkyilmaz et al. have indicated a negative correlation between low sACR and in-hospital mortality in patients with myocardial infarction following coronary procedures [11]. Low sACR is shown to be related to pulmonary infection in critical care [12]. In addition, sACR has been identified as a more effective biomarker for predicting adverse events in patients with cardiomyocyte injury than using albumin or creatinine alone [13,14]. This means that physicians can better assess the risk of potential complications in their patients and take necessary measures to prevent them. This result highlights the importance of using comprehensive biomarkers to monitor the health of patients and improve their outcomes.

Anthracyclines are chemotherapy drugs used to treat various types of cancer. However, despite their efficacy, they are also known to have a potent side effect of left ventricular dysfunction. Even low doses of anthracyclines ranging from 150 to 250 mg/m^2^ can cause both systolic and/or diastolic dysfunction in patients. Moreover, individuals receiving a cumulative doxorubicin dose of 350–400 mg/m^2^ are at a higher risk of experiencing cardiotoxicity due to anthracycline treatment. Several risk factors contribute to the development of anthracycline-induced cardiotoxicity. Pre-existing cardiovascular diseases such as coronary heart disease, hypertension, and hyperlipidemia, among others, predispose individuals to a higher risk of cardiotoxicity [15]. Additionally, concomitant cardiotoxic chemotherapy, obesity, sarcopenia, mediastinal radiation exposure, and older age are also significant risk factors for anthracycline-induced cardiotoxicity [16]. Therefore, healthcare providers must consider these and monitor patients closely to prevent or manage cardiotoxicity.

The development of cardiotoxicity can be attributed to several mechanisms, including the inhibition of protein synthesis, DNA damage, inflammation, and reactive oxygen species. Doxorubicin is known for the role it plays in producing reactive oxygen species (ROS), which are a key part of oxidative stress [17]. Research has shown that when doxorubicin produces too much ROS, it can lead to an increase in NF-κB expression, which in turn increases the inflammatory response [18]. These mechanisms can trigger inflammation, which is a critical factor in the damage of cardiomyocytes [19]. Cardiomyocyte damage and death set off an inflammatory cascade and oxidative stress, further damaging cardiac tissue. Inflammation plays a significant role in the progression of cardiac disease, as it can cause the death of healthy cells and contribute to the development of cardiotoxicity.

This study aims to evaluate the correlation between the serum albumin–creatinine ratio and the risk of anthracycline-induced cardiotoxicity in cancer patients.

## 2. Materials and Methods

### 2.1. Patients

We conducted a retrospective study of patients with cancer who received anthracycline-based chemotherapy in Hacettepe University Hospital between 2014 and 2023. Patients who had echocardiography and at least one follow-up echocardiography after the initiation of anthracycline treatment were included. Patients with no follow-up echocardiograms and patients with chronic kidney disease, acute infections, or inflammatory conditions that could affect the sACR values were excluded.

### 2.2. Data Collection

Data on patient demographics, comorbidities, underlying cancer and chemotherapy regimens, anthracycline type and doses, and baseline laboratory tests were collected for each patient from patient records. Cardiovascular disease was defined as the presence of hypertension, coronary heart disease, and diabetes mellitus. Total doxorubicin dose included the total dose administered to the patient both upfront and in subsequent lines of treatment if doxorubicin was re-administered. Reports of baseline (before anthracycline treatment) and follow-up echocardiographies were evaluated. Follow-up echocardiography is performed at the discretion of the oncologist in our institution, either based on symptoms or for periodical follow-up.

### 2.3. Definition of Anthracycline Cardiotoxicity

Cardiotoxicity was defined as the presence of systolic dysfunction according to LVEF criteria of the European Society of Cardiology (ESC) or diastolic dysfunction according to the criteria of the American Society of Echocardiography (ASE) [20,21].

### 2.4. Statistical Analysis

Statistical analyses were performed using SPSS V.24. All data are expressed as either median (IQR) for continuous variables or the number of patients (percentage) for categorical variables. Groups were compared using the chi-squares test for categorical variables and the Mann–Whitney U test or Kruskal–Wallis test for quantitative variables. The capacity of sACR, albumin, and creatinine values in predicting cardiotoxicity was analyzed using ROC curve analysis. The sensitivity and specificity were presented. Multivariable logistic regression analyses were performed to determine clinically relevant factors associated with the development of cardiotoxicity. Variables showing associations at a significance level of α = 0.20 in univariable analysis were selected for inclusion in the multivariable model. Covariates were also selected according to the results of previous research. Logistic regression analysis with backward selection was used for multivariate analysis. Hosmer Lemeshow goodness-of-fit statistics were used to assess model fit. A type-1 error level of <5% was used to infer statistical significance.

## 3. Results

### 3.1. Baseline Characteristics

The study included 525 patients after the exclusion of patients with chronic kidney disease or acute infectious or inflammatory conditions that could affect the sACR values (*n* = 15). Demographic and clinicopathologic characteristics of the study population stratified according to the sACR values are presented in Table 1. The median age was 49 (IQR = 40–59) years, 159 patients (30.3%) were men, and 366 patients (69.7%) were women. The median follow-up was 42 (IQR = 22–62) months. Breast cancer (49.9%) and lymphoma (37.1%) were the most common diagnoses. Cardiotoxicity developed in 82 patients (15.6%) The hemoglobin cut-off value (12.7 g/dL) was obtained by ROC analysis for the prediction of cardiotoxicity (AUC: 0.582%; 95 CI 0.52 to 0.65; *p*: 0.018).

### 3.2. Serum Albumin–Creatinine Ratio

ROC curve analysis was used for albumin, creatinine, and sACR to define the cut-off value. Among the three indices, sACR had the highest sensitivity, specificity, and AUC for predicting anthracycline-related cardiotoxicity (Table 2).

### 3.3. Relationship Between sACR and Anthracycline Cardiotoxicity

Patients were classified into sACR high and low subgroups according to ROC analysis results for the prediction of cardiotoxicity. The median doxorubicin dose was 236 mg/m^2^ (IQR = 218.9–251.9) and similar among patients with the sACR high group ± cardiotoxicity and those with sACR low group ± cardiotoxicity (*p* = 0.293; Table 3).

The results of the analysis are shown in Table 4. After adjusting for age, BMI, cardiovascular disease, hemoglobin, anthracycline dose, and gender, multivariate analysis revealed that sACR (HR = 1.85; 95% CI 1.12 to 3.06; *p* = 0.016), cardiovascular disease (HR = 1.97; 95% CI 1.08 to 3.61; *p* = 0.027), BMI (HR = 1.86; 95% CI 1.12 to 3.10; *p* = 0.017), and age (HR = 1.02; 95% CI 1.001 to 1.04; *p* = 0.036) were significantly associated with an increased risk of cardiotoxicity.

## 4. Discussion

To the best of our knowledge, this is the first study demonstrating a significant association between sACR and anthracycline-related cardiotoxicity. The results showed that patients with various cancers who were treated with anthracycline-containing chemotherapy had a higher risk of cardiotoxicity if their sACR level was low.

It is well known that both albumin and creatinine levels were significantly associated with increased chemotherapy toxicity and decreased overall survival [22,23]. However, the relationship between these markers and cardiotoxicity had not been previously assessed. The GeparOcto-GBG 84 trial, which was conducted in early-stage breast cancer patients only, examined creatinine levels and found a trend in creatinine’s univariate analysis (*p* = 0.07) [24]. In contrast, our study included patients with various cancers and found a relationship between the creatinine level and cardiotoxicity. This difference may be due to the heterogeneity of our study population.

Our research has shown that sACR is a promising indicator of cardiac dysfunction and can be closely related to malnutrition and muscle atrophy. Both albumin and creatinine are known to be markers for malnutrition and lower muscle mass, as well as key components of the systemic inflammatory response [25] that affects cardiac functions and overall survival after cardiomyocyte injury [26,27]. Consequently, the integrated evaluation of serum albumin and creatinine levels provides significant insights into the inflammatory status of patients.

In patients with myocardial injury, sACR serves as an important prognostic marker for long-term survival and is an independent predictor of cardiac events, including heart failure [13,28,29]. In addition, sACR is associated with hospital readmission in elderly patients with heart failure, which is closely related to quality of life and survival [30].Therefore, baseline sACR may be a valuable biomarker for identifying high-risk patients. However, it is important to acknowledge the potential impact of confounding variables on the results of sACR. These include but are not limited to liver and kidney function, cachexia, malnutrition, malabsorption syndromes, hormonal and metabolic factors such as thyroid dysfunction, diabetes, and obesity, which itself is a chronic inflammatory condition. Furthermore, even medications can affect albumin levels.

Overall, our results highlight the relationship between sACR and inflammation and cardiomyocyte injury. This information is relevant to clinicians and researchers who are investigating the role of biomarkers in predicting and diagnosing cardiac dysfunction. By identifying patients with high sACR levels, healthcare professionals can help prevent further cardiac damage and improve overall patient outcomes.

Anthracyclines are a type of chemotherapy that can cause drug-induced cardiotoxicity. This can lead to dysfunction in the heart’s systolic and/or diastolic function in 2.2% of patients. Since this damage is irreversible, it is crucial to identify patients who are at high risk of developing cardiotoxicity. Clinical risk factors include older age, obesity, sarcopenia, and cumulative anthracycline doses [15]. The most common way to detect cardiotoxicity is through baseline and follow-up monitoring of LVEF. However, global systolic longitudinal myocardial strain (GLS) is a more sensitive way to detect early injury [31]. A decrease in GLS compared to baseline is considered an early predictor of myocyte injury. Combining GLS and cardiac troponins can help detect cardiotoxicity in breast cancer patients [32]. This method has a 93% sensitivity and 91% negative predictive value. On the other hand, the SUCCOUR is a clinical trial that evaluates the use of GLS to guide patient management in cases of anthracycline-induced cardiotoxicity [33]. The trial found that GLS might be used to predict which patients will experience anthracycline related cardiotoxicity. However, cardiovascular events were rare, and thus the results of the trial had no impact on clinical practice. In addition to GLS, cardiac magnetic resonance imaging is not widely available in medical institutions, but it is also useful in detecting cardiac function alterations after chemotherapy. Blood-based biomarkers, such as troponin, are also used to assess cardiotoxicity. Various trials have demonstrated the predictive and prognostic value of troponins. However, the interpretation of data from troponin trials can be challenging due to differing thresholds, troponin subtypes, and testing times [34]. Similarly to GLS, troponins are not widely used to predict anthracycline induced cardiotoxicity. In a comparable manner to troponins and GLS, a number of trials including natriuretic peptides have yielded promising results in terms of predicting cardiotoxicity [35]. Conversely, the CARDIOTOX registry [36], which includes more than 800 patients, has demonstrated that baseline proBNP is not associated with cardiotoxicity. Finally, ongoing clinical trials are investigating blood-based inflammatory markers such as IL-6, Caspase-1, and myeloperoxidase to define the risk of drug-induced cardiotoxicity [37].

The sACR, which stands for the serum albumin-to-creatinine ratio, has major advantages when it comes to detecting cardiotoxicity in cancer patients. One of the most significant advantages is that it can be easily calculated without the need for any additional procedures, as most patients already undergo baseline laboratory tests that include albumin and creatinine measurements. This makes the process more convenient and less time-consuming for both patients and clinicians. Another advantage of using sACR is that it provides a baseline value that can help identify high-risk patients before chemotherapy is initiated, which can be extremely valuable in terms of the early detection and prevention of cardiotoxicity. In contrast, other methods such as monitoring a decrease in ejection fraction (EF) and global longitudinal strain (GLS) or an increase in cardiac biomarkers like BNP and troponins are generally considered only after treatment has already begun. Therefore, the earlier prediction of cardiotoxicity using the sACR might help clinicians take preventive measures or even switch to alternative treatment regimens, which can significantly reduce the risk of cardiotoxicity in cancer patients. Although there are conflicting results from cardioprotective trials, strategies have shown that the use of cardioprotective drugs such as ACE inhibitors, angiotensin receptor blockers, beta-blockers, or statins can effectively reduce the risk of cardiotoxicity in cancer patients [38,39]. In addition, ongoing clinical trials of neprilysin inhibitors and sodium–glucose co-transporter 2 inhibitors are exploring novel cardioprotective strategies [40]. Overall, the integration of the sACR into routine screening protocols would assist clinicians in identifying patients with a high risk of cardiotoxicity. The combination of laboratory and/or imaging-based biomarkers and clinical parameters would enhance both the specificity and sensitivity of the screening process.

It is important to consider the limitations of our study. Firstly, the retrospective study design may have impacted the results. Additionally, the patient population was heterogeneous, with varying stages of cancer. This study did not consider other inflammatory parameters, including pro-inflammatory cytokines, immunoglobulins, complement proteins, C-reactive protein (CRP), and cardiac markers such as troponins, creatine kinase-MB (CK-MB), and brain natriuretic peptide (BNP), as they are not routinely measured at our institution. Only the associations between the baseline sACR and cardiotoxicity were examined, without considering changes in the sACR over time. The study did not strictly define echocardiography indications and periods, which may have introduced biases. Early injury markers of cardiotoxicity, such as GLS and troponin, were not assessed. Furthermore, no association was found between the cumulative doxorubicin dose and the risk of cardiotoxicity, which could be due to the relatively lower doses used in most patients.

The results of this study indicate that the sACR is linked to a higher risk of anthracycline-related cardiotoxicity. Basic laboratory tests can easily assess the sACR before beginning anthracycline treatment. Patients with a high risk of cardiotoxicity should receive closer follow-up and cardioprotective measures.

## Figures and Tables

**Table 1:** Baseline Characteristics of patients treated with anthracycline therapy.

	All Patients (*n* = 525)	High sACR (*n* = 294)	Low sACR (*n* = 231)	Univariate Analysis *p* Value	Cardiotoxicity (+) (*n* = 82)
**Age (IQR)**	49 (IQR = 40–59)	47 (IQR = 39–55)	52 (IQR = 43–64)	<0.001	53 (IQR = 46–60)
**Gender** Male Female	159 (30.3%) 366 (69.7%)	39 (13.3%) 255 (86.7%)	120 (51.9%) 111 (%48.1)	<0.001	33 (40.2%) 49 (59.8%)
**Diagnosis** Breast Cancer Lymphoma Sarcoma Others *	262 (49.9%) 195 (37.1%) 35 (6.7%) 33 (6.3)	195 (66.3%) 67 (22.8%) 17 (5.8%) 15 (5.1%)	67 (29%) 128 (55.4%) 18 (7.8%) 18 (7.8%)	<0.001	33 (40.2%) 36 (43.9%) 6 (7.3%) 7 (8.5%)
**Chemotherapy Regimens** AC CAF CHOP İMA ABVD Others *	251 (47.8%) 11 (2.1%) 119 (22.7%) 28 (5.3%) 58 (11.0%) 58 (11.0%)	185 (62.9%) 9 (3.1%) 36 (12.2%) 14 (4.8%) 25 (8.5%) 25 (8.5%)	66 (28.6%) 2 (0.9%) 83 (35.9%) 14 (6.1%) 33 (14.3%) 33 (14.3%)	<0.001	31 (37.8) 2 (2.4%) 24 (29.3) 4 (4.9%) 7 (8.5%) 14 (17.1%)
**Body Mass İndex (kg/m^2^)** <25 ≥25	197 (37.5%) 328 (62.5%)	107 (36.4%) 187 (63.6%)	90 (39%) 141 (61%)	0.59	37 (45.1%) 45 (54.9%)
**Anthracycline Dosage (mg/m^2^) (IQR)**	236 (IQR = 218.9–251.9)	235.3 (IQR = 223.5–241.8)	238,1 (IQR = 198.9–291.7)	0.29	234.6 (IQR = 191.6–246.8)
**Hemoglobin (g/dL)** <12.7 ≥12.7	224 (42.7%) 301 (57.3%)	116 (39.5%) 178 (60.5%)	108 (46.8%) 12.3 (53.2%)	0.11	44 (53.7%) 38 (46.3%)
**Cardiovascular Disease** Yes No	88 (16.8%) 437 (83.2%)	50 (%17) 244 (83%)	38 (16.5%) 193 (83.5%)	0.91	22 (26.8%) 60 (73.2%)
**Albumin (g/dL)** <4.09 ≥4.09	251 (47.8%) 274 (52.2%)	101 (34.4%) 193 (65.6%)	150 (64.9%) 81 (35.1%)	<0.001	45 (54.9%) 37 (45.1%)
**Creatinine (mg/dL)** <0.7 ≥0.7	286 (54.5%) 239 (45.5%)	249 (84.7%) 45 (15.3%)	37 (16%) 194 (84%)	<0.001	36 (43.9%) 46 (56.1%)

* Bone/soft tissue sarcoma, thymic cancer, uterine cancer, multiple myeloma, hepatocellular carcinoma, chronic lymphocytic leukemia. ABVD, doxorubicin–bleomycin–vinblastine–dacarbazine; AC, doxorubicin–cyclophosphamide; CAF, cyclophosphamide–doxorubicin–fluorouracil; CHOP, cyclophosphamide–doxorubicin–vincristine—prednisone; İMA, ifosfamide–mesna–doxorubicin.

**Table 2:** Sensitivity, specificity, and area under curve values of albumin, creatinine, and sACR for the prediction of cardiotoxicity.

	Sensitivity	Specificity	Cut-Off Value	Area Under Curve	*p* Value
**Albumin (g/dL)**	54.9	53.5	4.09	0.568 (%95 CI 0.50 to 0.64)	0.052
**Creatinine (mg/dL)**	56.1	56.4	0.70	0.599 (%95 CI 0.53 to 0.67)	0.004
**sACR**	58.5	58.9	5.79	0.633 (%95 CI 0.56 to 0.70)	<0.001

**Table 3:** Median doxorubicin doses in patients according to sACR high or low group and cardiotoxicity.

	Cardiotoxicity (+)	Cardiotoxicity (−)
**sACR high**	234.6 mg/m^2^	235.3 mg/m^2^
**sACR low**	235.3 mg/m^2^	239.1 mg/m^2^

Kruskal–Wallis, *p* = 0.293; sACR: serum albumin–creatinine ratio.

**Table 4:** Univariate and multivariate analysis of covariates associated with the development of cardiotoxicity.

	Univariate Analysis *p* Value	Multivariate Analysis HR (95% CI) *p* Value
Age, years	0.005	HR = 1.02% 95 CI 1.001 to 1.04 *p* = 0.036
Gender Female Male	0.033	HR = 0.77% 95 CI 0.44 to 1.35 *p* = 0.359
**Diagnosis** Breast Cancer Lymphoma Sarcoma Others *	0.28	(−)
**Albumin (g/dL)** <4.09 ≥4.09	0.163	
Creatinine (mg/dL) <0.7 ≥0.7	0.036	
sACR <5.79 ≥5.79	0.002	HR = 1.85% 95 CI 1.12 to 3.06 *p* = 0.016
Cardiovascular Disease No Yes	0.008	HR = 1.97% 95 CI 1.08 to 3.61 *p* = 0.027
Body Mass İndex <25 ≥25	0.122	HR = 1.86, 95% CI 1.12 to 3.10, *p* = 0.017
**Anthracycline Dosage (mg/m^2^)**	0.144	HR = 0.998% 95 CI 0.995 to 1.002 *p* = 0.292
**Hemoglobin (g/dL)** <12.7 ≥12.7	0.028	HR = 1.51% 95 CI 0.92 to 2.50 *p* = 0.104

* Bone/soft tissue sarcoma, thymic cancer, uterine cancer, multiple myeloma, hepatocellular carcinoma, chronic lymphocytic leukemia.

## Data Availability

Data are available upon reasonable request. The data that support the findings of this study are available from the corresponding author upon reasonable request.
